# Accelerated decline in lung function in smoking women with airway obstruction: SAPALDIA 2 cohort study

**DOI:** 10.1186/1465-9921-6-45

**Published:** 2005-05-26

**Authors:** Sara H Downs, Otto Brändli, Jean-Pierre Zellweger, Christian Schindler, Nino Künzli, Margaret W Gerbase, Luc Burdet, Robert Bettschart, Elisabeth Zemp, Martin Frey, Roland Keller, Jean-Marie Tschopp, Philippe Leuenberger, Ursula Ackermann-Liebrich, the SAPALDIA team

**Affiliations:** 1 Institute of Social and Preventive Medicine, University of Basle, Basle, Switzerland; 2 Zürcher Höhenklinik, Wald, Switzerland; 3 Service of Pulmonology, University Hospital Lausanne, CHUV, Switzerland; 4 Division of Occupational and Environmental Health, University of Southern California, USA; 5 Division of Pulmonary Medicine, University Hospitals of Geneva, Geneva, Switzerland; 6 Hôpital intercantonal de la Broye, Payerne, Switzerland; 7 Hirslanden Klinik Aarau, Switzerland; 8 Klinik Barmelweid, Aarau, Switzerland; 9 Centre Valaisan de Pneumologie, Montana, Switzerland

## Abstract

**Background:**

The aim was to determine if effects from smoking on lung function measured over 11 years differ between men and women.

**Methods:**

In a prospective population based cohort study (Swiss Study on Air Pollution and Lung Diseases in Adults) current smokers in 1991 (18 – 60 yrs) were reassessed in 2002 (n = 1792). Multiple linear regression was used to estimate effects from pack-years of cigarettes smoked to 1991 and mean packs of cigarettes smoked per day between 1991 and 2002 on change in lung volume and flows over the 11 years.

**Results:**

In both sexes, packs smoked between assessments were related to lung function decline but pack-years smoked before 1991 were not. Mean annual decline in FEV_1 _was -10.4 mL(95%CI -15.3, -5.5) per pack per day between assessments in men and -13.8 mL(95%CI-19.5,-8.1) in women. Decline per pack per day between 1991 and 2002 was lower in women who smoked in 1991 but quit before 2002 compared to persistent smokers (-6.4 vs -11.6 mL, p = 0.05) but this was not seen in men (-14.3 vs -8.8 mL p = 0.49). Smoking related decline was accelerated in men and women with airway obstruction, particularly in women where decline in FEV_1 _was three fold higher in participants with FEV1/FVC<0.70 compared to other women (-39.4 vs -12.2 mL/yr per pack per day, p < 0.002).

**Conclusion:**

There are differences in effects from smoking on lung function between men and women. Lung function recovers faster in women quitters than in men. Women current smokers with airway obstruction experience a greater smoking related decline in lung function than men.

## Background

Cigarette smoking is the most well known risk factor for accelerating lung function decline in adults [1]. Until recently, smoking prevalence and intensity was greater in men than in women; but there is now evidence that women are starting to smoke as much as men [1,2]. At present, there is no consensus whether women are more sensitive to effects from cigarette smoke than men [1-8]. Relatively few large studies have compared effects from smoking between men and women and results have not been consistent between studies. A greater decline in lung function per pack-year in women compared to men was reported by Chen *et al *but the converse was found by Xu *et al *[5,6]. Prescott *et al *reported slightly higher coefficients for decline in FEV_1 _per pack-year in women compared to men and Connett *et al *reported similar rates of decline in men and women but a swifter regain in FEV_1 _in women who quit smoking [4,7].

Smoke related lung damage is characterised by inflammation, airway obstruction and destruction of the lung parenchyma [9,10]. Underlying lung characteristics can vary and there is some evidence that smokers differ in their predisposition to develop predominantly emphysema or bronchitis [11,12]. Predispositions between men and women may vary because of differences in lung morphology that modify the dispersion and deposition of cigarette smoke or differences in homeostatic processes affecting the efficacy of lung clearance and recovery after smoking cessation [13-15].

The strongest effects from smoking have been consistently measured in current smokers and detrimental effects have been shown to reduce with time from cessation [1,16]. The vast majority of surveys of effects from smoking on lung function have focused on FEV_1 _(forced expiratory volume in one second). However, effects may be also be revealed in other measures of lung function such as FEF_25 _(forced expiratory flow at 25% of lung volume) that may better reflect damage to the small airways. An argument for not investigating lung flow rates has been the large inter-subject variability in the rates which prejudices against precise estimation of effects in cross-sectional analyses. In a cohort study however, the inter-participant differences become less important because each subject serves as its own control.

The Swiss Study on Air Pollution and Lung Diseases in Adults (SAPALDIA) is a prospective population based cohort study initiated in 1991 and designed to measure long term effects of air pollution and other risk factors on respiratory health [17]. In cross-sectional analyses, associations were found between levels of lung function and exposure to air pollutants as well as to cigarette smoke [18-20]. Eleven years later, participants have been re-examined using the same methodology. Lung volumes and flows as well as smoking history were measured in 1991 and 2002. The aim of the present analysis is to assess the long term effects from smoking on change in lung function and to compare effects between men and women. The sample examined is current smokers at the time of the first assessment (1991). Effects from smoking up to 1991 and between 1991 and 2002 (SAPALDIA 1 and 2) are compared for lung flows (FEF_25_, FEF_50 _and FEF_75_) as well as for FEV_1_.

## Methods

SAPALDIA is a multi-centre population based prospective cohort study. The study participants were recruited from random population samples from local registries of inhabitants from eight areas of Switzerland. The eight areas were chosen to represent the variety of environmental conditions found in Switzerland concerning geography, climate, degree of urbanisation and air pollution. To meet the selection criteria, individuals had to be 18 to 60 years of age by December 1990, local residents for at least three years and Swiss nationals or foreigners with a residence permit. There were 9651 participants in SAPALDIA 1 representing 60% of the eligible sample. Participants in SAPALDIA 1 were invited for re-examination between 2001 and 2003. Methods in SAPALDIA have been described in detail [17,21]. Ethical approval for the study was given by the Regional Ethics Commission for Clinical Medicine (Swiss Academy of Medical Sciences) and each centres' regional ethics committee.

Identical protocols for spirometry that complied with American Thoracic Society recommendations, were followed in SAPALDIA 1 and 2 [22,23]. The highest values for forced vital capacity (FVC) and FEV_1 _of an acceptable trial were selected. Measures of expiratory flows (FEF_75_, FEF_50 _and FEF_25_) were taken from the flow-volume curve with the highest sum of FVC and FEV_1_. Participants were requested not to use beta-2-agonists or anticholinergic inhalers four hours prior to and long-acting beta agonists, oral beta-2-agonists, theophyline or oral antimuscarinic medication eight hours prior to the time of appointment of the examination.

Information about smoking and other risk factors was gathered through an interview administered questionnaire based on the European Community Respiratory Health Survey (ECRHS) questionnaire [24]. Three categories of smoking status were derived: current smoker, former smoker and never smoker. Smokers had to have smoked more than 20 packs of cigarettes or more than 360 g of tobacco. 'Current' smokers were smokers who had smoked within the month before the interview. Other participants with validated smoking histories were classified as former smokers. Cumulative cigarette smoking exposure was summarised into two variables: pack-years to SAPALDIA 1 based on responses at SAPALDIA 1 and mean packs of cigarettes smoked per day between SAPALDIA 1 and 2 (packs per day) based on responses at SAPALDIA 1 and 2. Pack-years were calculated as years of smoking multiplied by number of cigarettes per day divided by 20.

Participants were asked not to smoke in the hour before the examination and this was validated in both surveys by measuring carbon monoxide (CO) concentration in exhaled breath using a EC 50 Micro-Smokerlizer Bedfont measuring device.

### Statistical analysis

The effect of smoking on mean annual change in FEV_1_, FEF_25_, FEF_50 _and FEF_75 _between surveys was analysed by multiple linear regression. The main covariates investigated were pack-years to SAPALDIA 1 and mean packs per day smoked between surveys. All analyses were confined to subjects classified as current smokers at SAPALDIA 1.

Separate models analyses were conducted for men and women. Covariates tested in the regression models, other than the variables "pack-year" and "packs per day" included study area, atopy, childhood respiratory infection before age five, maternal smoking, paternal smoking, education, current and ever exposure to dust and fumes at work, age at start of smoking, inhaler or not, pipe smoker, solely non-cigarette smoker, body mass index (BMI), weight at baseline, change in weight, change in BMI between surveys and years since quitting. Predictors in each model are shown in the footnote to table 3. Baseline lung function parameters were included in all models because we could not assume that the change in lung function would be the same for all lung volumes and because we wanted to absorb effects from smoking up to baseline (SAPALDIA 1). However, effects were also re-examined in models that did not contain baseline lung function covariates. For each continuous covariate, we tested whether a linear, quadratic or cubic polynomial best described the relation with change in lung function. Statistical tests for interaction were conducted to determine differences in effects from smoking between men and women and between subjects with FEV_1_/FVC greater or less than 0.70.

Fourteen men and 11 women were excluded from the regression analyses because they had been classified as current smokers at SAPALDIA 1 and claimed to be never smokers at SAPALDIA 2. Five men and three women were excluded because they claimed to be former smokers at SAPALDIA 2 but had expired CO concentrations greater than 10 ppm (median 15.5, IQR 11.5–20). There were 53 women and 99 men respectively for whom information on pack-years or packs smoked were missing and 74 participants with missing flow data.

Regression diagnostics were conducted to identify influential data. Sensitivity analyses were conducted to examine effects after eliminating points with high leverage or large residuals and to assess possible biases due to missing information about numbers of cigarettes smoked. Analyses were conducted using STATA SE version 8.0 (StataCorp, Texas, 77845 USA).

## Results

Of the 3232 current smokers in 1991 at SAPALDIA 1, 1792 (55%) participants provided spirometry and smoking information from both surveys (see Table 1). Smokers who did not participate in SAPALDIA 2 had slightly worse lung function at baseline. Male smokers were more likely than women to show evidence of airway obstruction (FEV_1_/FVC ratio<0.70) than female smokers and had accumulated more pack-years. The period of follow-up was the same in men and women (median 10.9 years, IQR 10.8–11.0).

**Table 1:** Characteristics of current smokers in 1991 (SAPALDIA 1)

	Participants in SAPALDIA 1 and 2*				Participants only in SAPALDIA 1 †			
Characteristic measured in 1991	Men (n = 978)		Women (n = 814)		Men (n = 844)		Women (n = 596)	

Median age [yrs] (IQR)	41.8	(32.1, 49.6)	38.4	(31.0, 46.6)	40.2	(30.7, 49.2)	39.8	(30.8, 47.3)
Mean height [cm] (SD)	175.2	(6.8)	163.2	(6.6)	174.4	(7.2)	163.4	(6.5)
Mean weight [kg] (SD)	76.7	(11.1)	59.6	(9.5)	75.4	(11.7)	61.8	(13.8)
% atopic	23.2		18.4		22.6		22.5	
% severe respiratory infection as an infant	6.2		9.5		5.3		6.7	
% basic schooling only	13.8		17.7		22.1		22.1	
% exposed to dust & fumes at work	45.5		25.1		48.2		25.2	
% mother smoked	13.4		18.3		14.4		22.4	
% father smoked	61.3		58.5		63.1		59.9	
Median cig/day (IQR)	20	(14, 30)	20	(10, 20)	20	(15, 30)	20	(10, 23)
Median pack-years (IQR)	20.6	(8.3, 36.1)	14.4	(6.4, 25.0)	21.3	(9.1, 38.2)	15.9	(7.2, 26.7)
% inhaler	82.9		88.8		87.9		85.9	
Median age started smoking [yrs] (IQR)	18	(16, 20)	18	(16, 20)	18	(16, 20)	18	(16, 20)
Median carbon monoxide [ppm] (IQR)	19	(9, 30)	16	(8, 28)	24	(13, 35)	20	(10, 30)
Mean FVC [mL] (SD)	5238	(861)	3868	(624)	5079	(874)	3745	(631)
Mean FEV_1_[mL] (SD)	4031	(759)	3099	(552)	3902	(837)	2957	(575)
Mean FEF_25_[mL/s] (SD)	1535	(793)	1427	(748)	1562	(871)	1325	(734)
Mean FEF_50_[mL/s] (SD)	4535	(1529)	3807	(1127)	4421	(1641)	3600	(1182)
Mean FEF_75_[mL/s] (SD)	7876	(2133)	5816	(1479)	7528	(2370)	5536	(1516)
% FEV_1_/FVC≤0.7	15.9		9.3		19.8		14.1	

*With information on spirometry and smoking

† Includes participants in SAPALDIA 2 who provided information on smoking but no spirometry IQR = inter-quartile range

Approximately 30 % of men and women who were current smokers in 1991 had quit by 2002. Men had accumulated more pack-years between surveys but were more likely to have quit smoking (see Table 2). Median years since quitting amongst quitters were 5.5 (IQR 2.1 to 9.7) in men and 4.2 (IQR 2.2 to 9.2) in women. The correlation coefficient between pack-years smoked before 1991 and pack-years smoked between surveys was 0.55 in men and 0.68 in women. Mean weight change was +5.4 kg in men and +5.5 kg in women. Absolute mean declines in lung function were greater in men compared to women except for FEF_25_. After controlling for mean lung size or flow measured in 1991 differences in change between men and women became less significant except for FEF_25_. Mean percent change in FEF_25 _from baseline was -3.4 (5%CI -3.6, -3.2) in women and -2.8 (95%CI -3.0, -2.7) in men. The greatest inter-subject variability in change was observed for FEF_75 _(forced expiratory flow at 75% of lung volume).

**Table 2:** Smoking and lung function measured in 2002 (SAPALDIA 2)

	Men n = 978		Women n = 814	
% new former smokers	31.7		27.2	
Median pack-years between 1991 and 2002 (IQR)	9.0	(3.8, 13.5)	7.6	(4.0, 11.0)
% inhaler	84.7		90.3	
Mean annual change in:				
FVC [mL] (SD)	-32.6	(48.9)	-20.7	(35.8)
FEV_1_[mL] (SD)	-43.8	(36.6)	-34.7	(27.2)
FEF_75_[mL/s] (SD)	-59.1	(167.4)	-34.1	(121.6)
FEF_50_[mL/s] (SD)	-78.7	(90.9)	-70.1	(71.9)
FEF_25_[mL/s] (SD)	-46.9	(47.3)	-51.8	(45.7)

**Table 3:** Mean annual change in lung function per pack-year to 1991 and per pack of cigarettes smoked per day between 1991 and 2002 in women and men

	Term	Annual change per pack-year to 1991			Annual change per packs per day between 1991 and 2002		
							
		Coef	95%CI	p value	Coef	95%CI	p value

FEV_1_[mL]							
Men (n = 840)	Linear	-0.1	-0.2, 0.1	0.50	-10.4	-15.3, -5.5	<0.001
Women (n = 735)	Linear	1.3	0.7, 2.0	<0.001	-13.8	-19.5, -8.1	<0.001
	Quadratic	-0.04	-0.06, -0.02	<0.001	na	na	na
	Cubic	0.0004	0.0002, 0.0005	<0.001	na	na	na
FEF_75_[mL/s]							
Men (n = 798)	Linear	-0.6	-1.4, 0.1	0.11	-35.1	-57.5, -12.7	0.002
Women (n = 697)	Linear	-0.2	-1.0, 0.7	0.64	-18.6	-42.7,4.9	0.12
FEF_50_[mL/s]							
Men (n = 825)	Linear	-0.2	-0.6, 0.2	0.31	-22.4	-34.5, -10.3	<0.001
Women (n = 697)	Linear	0.2	-0.3, 0.8	0.31	-31.3	-46.1, -16.5	<0.001
FEF_25_[mL/s]							
Men (n = 823)	Linear	-0.05	-0.1, 0.1	0.59	-8.9	-14.5, -3.4	<0.002
Women (n = 731)	Linear	1.1	0.2, 1.9	0.01	-10.0	-17.3, -2.8	0.007
	Quadratic	-0.03	-0.6, -0.01	0.01	na	na	na
	Cubic	0.0003	0.00005, 0.0005	0.006	na	na	na

na: no association with polynomial terms

Covariates other than smoking variables:

Models for Men:

FEV_1 _FEV_1_, FEV_1_^2^, age, height, atopy, weight change, current exposure to dust & fumes at work, study area

FEF_75 _FEF_75_, FEF_75_^2^, age, age^2^, atopy, maternal smoking, study area

FEF_50 _FEF_50_, age, weight, study area

FEF_25 _FEF_25_, FEF_25_^2^, FEF_25_^3^, age, weight, weight change, study area

Models for Women:

FEV_1 _FEV_1_, FEV_1_^2^, age, age^2^, height, height^2^, atopy, weight change, study area

FEF_75 _FEF_75_, FEF_75_^2^, FEF_75_^3 ^age, paternal smoking, study area

FEF_50 _FEF_50_, age, weight, weight change, paternal smoking, study area

FEF_25 _FEF_25_, age, age^2^, height, weight, weight change, study area

The adjusted effects from pack-years of cigarettes smoked to 1991 and per pack smoked per day per year between 1991 and 2002 are shown in table 3. Removal of baseline lung function variables from models made only small differences to effect estimates. Point estimates for effects from pack-years were stable in all models for both men and women after removal of influential observations.

In men, there was no association between pack-years to 1991 and change in lung function between 1991 and 2002 (see Table 3). The covariate for pack-year was retained as a linear term in all models however, because it influenced effect estimates for packs per day between 1991 and 2002. Restricting models to participants who were current smokers in both surveys made no difference to the effect estimates for pack-years to 1991.

In contrast to men, significant non-linear relations between annual change in lung function and pack-years smoked to 1991 were found in women (see Tables 3, 4 and 5). The polynomial terms were highly significant. A non-linear relation between pack-years and change in FEF_25 _was also seen in women that was not seen in men.

**Table 4:** Mean annual change in FEV_1 _per pack-year to 1991 and per pack of cigarettes smoked per day between 1991 and 2002 in quitters* and persistent smokers

	Term	Annual change in mL per pack-year to 1991			Annual change in mL per packs per day between 1991 and 2002		
		Coef	95%CI	p value	Coef	95%CI	p value

Quitters†							
Men (n = 245)	Linear	-0.1	-0.4, 0.3	0.78	-14.3	-22.6, -6.0	0.001
Women (n = 179)	Linear	1.5	0.3, 2.6	0.01	-6.4	-17.6, 4.6	0.25
	Quadratic	-0.04	-0.08,-0.01	0.01	na	na	na
	Cubic	0.0004	0.0001,0.0006	0.006	na	na	na
Persistent smokers†							
Men (n = 595)	Linear	-0.1	-0.3, 0.12	0.41	-9.0	-15.4, -2.5	0.006
Women (n = 556)	Linear	1.4	0.5, 2.3	0.001	-11.6	-19.8, -3.5	0.005
	Quadratic	-0.06	-0.08, -0.02	<0.001	na	na	na
	Cubic	0.0006	0.0003, 0.0008	<0.001	na	na	na

*Quitters were current smokers in 1991 who had stopped smoking by 2002.

† P values for interaction between persistent smoking and quitting with packs per day: men = 0.49 women = 0.05

na: no association with polynomial terms

Effects estimated from regression models with same covariates as in Table 3

**Table 5:** Mean annual change in FEV_1 _per pack-year to 1991 and per pack of cigarettes smoked per day between 1991 and 2002 by degree of airway obstruction in 1991

	Term	Annual change in mL per pack-year to 1991			Annual change in mL per packs per day between 1991 and 2002		
		Coef	95%CI	p value	Coef	95%CI	p value

FEV1/FVC>0.7†							
Men (n = 692)	Linear	-0.1	-0.3, 0.1	0.48	-8.8	-14.1, -3.5	0.001
Women (n = 659)	Linear	1.4	0.8, 2.0	<0.001	-12.2	-18.1, -6.4	<0.001
	Quadratic	-0.04	-0.06, -0.03	<0.001	na	na	na
	Cubic	0.0004	0.0002, 0.0005	<0.001	na	na	na
FEV1/FVC<0.7†							
Men (n = 127)	Linear	-0.2	-0.6, 0.2	0.36	-12.9	-25.0, -0.7	0.04
Women (n = 67)	Linear	-0.9	-2.2, 0.5	0.21	-39.4	-69.1, -9.6	0.01
	Quadratic	0.02	0.01, 0.04	0.009	na	na	na

† P values for interaction between high and reduced FEV1/FVC with packs per day: men = 0.07 women = 0.002

na: no association with polynomial terms

Effects estimated from regression models with same covariates as in Table 3

Lung function decline was strongly associated with packs of cigarettes smoked per day between 1991 and 2002 in both men and women (see Table 3). Point estimates were of a similar magnitude in both sexes with an additional mean annual decline in FEV_1 _per pack of cigarettes smoked per day of -10.4 mL in men and -13.8 mL in women. Similar patterns to those seen for FEV1, of stronger effects from recent smoking than from past, were found in the change in flows and especially for FEF_25 _(forced expiratory flow at 25% of lung volume).

Whereas the decline in FEV_1 _per pack per day smoked between surveys was smaller in women quitters compared to women continuing smokers; there was no difference in effect estimates for men (see Table 4) (p = 0.12 for difference in effect of pack-years in quitters between men and women). Inclusion of a variable for years since quitting into the models for quitters reduced effect estimates for change in FEV1 per pack per day to -13.4 mL/yr (95%CI -1.9 to 2.2) in men and -0.7 mL/yr (-0.6 to 1.9) in women thereby magnifying the difference in effects between men and women quitters (p = 0.07). Difference in effect estimates between women persistent smokers and quitters was also increased by adjusting for years since quitting (p = 0.02).

**Figure 1:**
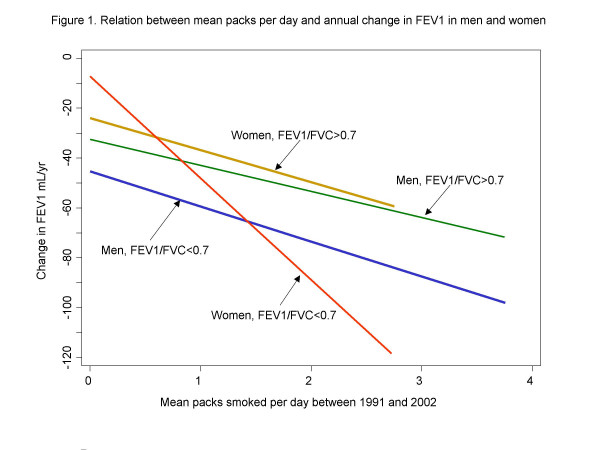
Relation between annual decline in FEV_1 _and mean packs/day smoked between 1991 and 2002 from regression models reported in Table 3

Decline in lung function per pack per day between surveys was greater in men and women with a reduced FEV_1_/FVC at baseline compared to other participants (see Table 5 and Figure 1). The difference in effect per packs per day on annual change in FEV_1 _between women with and without FEV_1_/FVC <0.70 was three fold and highly significant (p < 0.002). A smaller difference in the effect from cigarette smoking between men with and without FEV_1_/FVC <0.70 was seen (p = 0.07). The p value for the difference in effects between men and women with a reduced FEV_1_/FVC was 0.05.

## Discussion

Using quantitative information on cigarettes smoked during two time periods, we show similarities and differences in the effect of smoking on lung function decline between men and women. Decline due to smoking is strongly related to recent exposure and to a similar magnitude in men and women overall. However, we found evidence that the facility for recovery from past smoking is greater in women quitters. In both men and women, effects from recent smoking were more detrimental to lung function in individuals with pre-existing airway obstruction. However, the strongest effects from smoking were seen in the additional decline in FEV_1 _in women with a reduced FEV_1_/FVC ratio.

The mean unadjusted changes in lung function over the 11-year follow-up of the SAPALDIA cohort study are broadly consistent with changes reported elsewhere [6,25,26]. Average annual declines in FEV_1 _in SAPALDIA were higher than those for smokers in the ECRHS although lower than those reported in the Lung Health Study. Mean annual declines in FEV1 were -35 mL in men and -27 mL in women persistent smokers in ECRHS but participants were on average younger than the SAPALDIA participants (mean age 34 years versus 40 at study entry). Mean annual declines of -66 ml and -54 mL in FEV_1 _were reported for men and women continuing smokers respectively in the Lung Health Study (LHS). However, the LHS participants were older (35–60 years versus 18–60 years), had chronic obstructive airways disease and also reported heavier smoking (mean of 30 cigarettes per day versus 20 in SAPALDIA) at study entry [26]. In the Six Cities study, effect estimates for mean annual change in FEV_1 _per pack smoked in men (-12.6 ml per pack per day, 95%CI -9.7, -15.5) were similar to the effects we found in men but estimates in women (-7.2 ml per pack per day, 95%CI -9.5, -4.8) were lower than ours. However, the effects of smoking prior to follow-up and smoking during the follow-up period (3–6 years) were summarized into one variable of packs smoked and were not examined separately in the Six Cities study [6].

In both men and women, mean packs smoked per day between 1991 and 2002 were linearly associated with decline in lung function over the time period but pack-years to 1991 were not. The point estimates for annual change in lung function per pack per day in the most recent 11 years were similar in men and women with no evidence of airway obstruction. There was some evidence that men and women with poor lung function and who smoked heavily were less likely to participate in the second survey and this may be part of the explanation why we did not measure a negative effect from earlier pack-years.

Pack-years of cigarettes smoked before the 1991 were associated with non-linear changes in FEV_1 _and FEF_25 _between surveys in women. The polynomial terms were highly significant (p < 0.001) implying multiple testing is unlikely to be an explanation. Possibly women, as a group, have a more heterogeneous response to smoking than men. We found evidence that suggested that women both recover more swiftly after quitting smoking than men but also that women with airway obstruction are more vulnerable to effects from smoking.

The distribution of years since quitting amongst men and women quitters was similar and relatively short. Previous studies have also reported attenuated effects from smoking after short periods of cessation [7,27,28]. In the LHS, the attenuation in lung function decline following smoking cessation could be observed after one year [7]. The significance of the difference in effect from packs smoked per day between women quitters and persistent smokers was marginal (p = 0.05), but increased with adjustment for years since quitting; consistent with a recovery effect related to quitting. We did not observe an attenuated effect on lung function decline following smoking cessation in men, which has been reported elsewhere [7,29]. However, the number of quitters in our study was relatively small and in the Tucson Epidemiological Study of Airways Obstructive Disease, recovery in men was restricted to young men with no evidence of airway obstruction [29]. Our data provide additional evidence that facility for recovery following smoking cessation is, overall, greater in women compared to men.

Reliability of reporting habits may differ between sexes and we are unable to assess the possible extent of this bias in our study. However, we attempted to validate smoking status using expired CO and similar proportions of men and women were shown to have reported of inconsistent information about smoking habits. In the ECRHS women were as likely as men to report cough and phlegm but significantly less likely to have airflow obstruction [30]. If perception of decline in lung function is greater in women than in men, susceptible women may be more likely to quit smoking than susceptible men.

Evidence for a larger effect from smoking in men with symptoms of obstructive airway disease was presented by Fletcher and Peto almost 30 years ago [31]. Our data showed that both women and men with a reduced FEV_1_/FVC experienced an accelerated decline in FEV_1 _associated with recent cigarette smoking but the effect was significantly greater in women. We are unaware of other studies showing an accelerated effect in lung function decline related to smoking in women with obstructed airways compared to men. However, the female predominance in the Boston Early-Onset COPD Study has been attributed to a higher risk for the development of severe COPD in women compared to men [32]. Men and women persistent smokers in the LHS study have been reported to be approximately equivalent in terms of their percent predicted lung function loss [7]. However, since all LHS participants had evidence of mild to moderate airway obstruction at study entry, we would predict a sex difference in lung function decline per pack of cigarette smoked.

Reduced flow rates are due to a combination of airway narrowing and decreased lung recoil [9]. Predisposition to airway narrowing and decreased lung recoil may vary between men and women given sex differences in lung characteristics [15]. In addition, experimental evidence suggests that the distribution of particle deposition in the airways is likely to be more proximal in women compared to men [14]. Since airway caliber is smaller in women, we could hypothesise that the same reduction in airway diameter would result in a relatively greater impact on the reduction in flow rates in women compared to men [15,33].

An accelerated rate of lung function decline has also been reported amongst asthmatics who smoke [34]. Excluding women with bronchial hyperresponsiveness from our population sample did not reduce the estimates for the effect of packs/day on decline in FEV_1 _(data not shown). Therefore, although asthmatics may well have experienced an accelerated lung function decline it seems unlikely that asthma per se is an explanation for the different rates of decline between men and women with a reduced FEV_1_/FVC. Our findings suggest that smokers with pre-existing airway obstruction are likely to experience accelerated lung function decline irrespective of the potential underlying disease.

## Conclusion

Some of the inconsistent findings from earlier studies comparing effects from smoking on lung function between men and women may be due to difficulties in separating out effects from recent smoking and past smoking. In both men and women recent smoking is a much stronger predictor of lung function decline than smoking more than 10 years previously. Our findings suggest that women recover faster from past smoking than men, but are particularly susceptible to effects from current smoking when they have existing airways obstruction. This observational study over 11 years suggests that women should have even greater incentives, in terms of lung function, to quit smoking than men.

## Competing interests

The author(s) declare that they have no competing interests.

## Authors' contributions

SD conducted the analyses and drafted the article. OB, JPZ, CS, NK, MG, LB, RB, EZ, MF, RK JMT, UA and PL contributed to the design of the study, the acquisition of data and the interpretation of data. CS also advised on the conduct of the analyses. All authors contributed to the conception of the research question, made important intellectual contributions during the drafting process and have given approval for the final version.
